# Usual interstitial pneumonia

**DOI:** 10.11604/pamj.2022.42.92.35264

**Published:** 2022-06-03

**Authors:** Divya Manoj Badjate, Moli Jain

**Affiliations:** 1 Department of Cardio-Respiratory Physiotherapy, Ravi Nair Physiotherapy College, Datta Meghe Institute of Medical Sciences (DU), Wardha, Maharashtra, India

**Keywords:** Usual interstitial pneumonia, honeycombing pattern, tractional bronchiectasis, high-resolution computed tomography

## Image in medicine

This clinical scenario includes a male, aged 83 years, a retired railway worker with complaints of breathlessness (modified Medical Research Council scale grade V), cough with expectorations (thick yellow-colored), and chest pain (sudden in onset, gradually progressive, and retrosternal in nature). He was a known case of hypertension for 15 years on medications, tuberculosis 10 years back for which he was managed with antitubercular treatment. He also suffered from a cerebrovascular event 4 years back and is currently on medications. On examination, the chest was a barrel-shaped and tracheal deviation to the right side. The movements of the chest wall were diminished bilaterally, more diminished on the left side along with the use of accessory muscles of the respiration with reduced chest expansion. On percussion, the dull note was present over lower intercostal spaces bilaterally. On auscultation, air entry on bilateral lung fields was reduced, with crepitations present on lower zones bilaterally. High-resolution computed tomography (HRCT) scan of the thorax was done which revealed - tractional bronchiectasis and multiple fibrotic changes in bilateral lung fields showing apicobasal gradient (A and C), with honeycombing pattern (D) and patchy areas of consolidation and thickening of the septa in bilateral lung fields (B). Findings are suggestive of the Usual Interstitial Pneumonia (UIP) pattern. He was advised medical management along with physiotherapy rehabilitation, which specifically included palliative care and supervised pulmonary rehabilitation.

**Figure 1:**
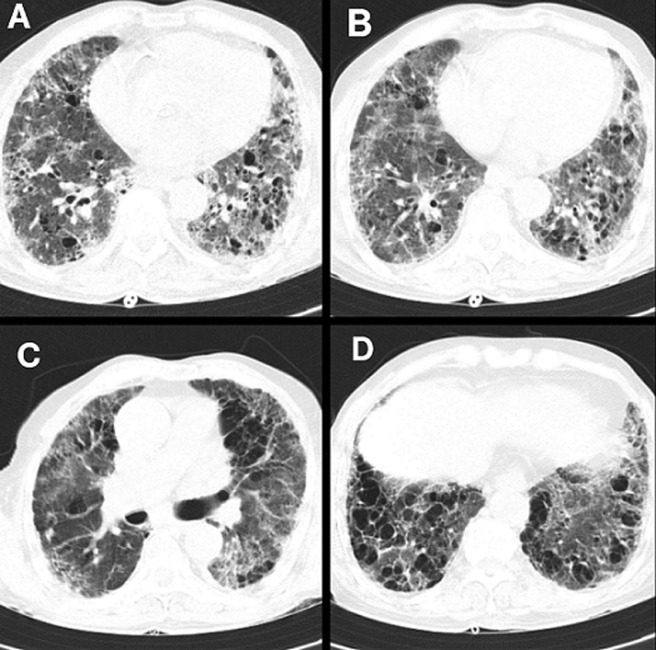
HRCT scan of the thorax, (A and C) tractional bronchiectasis and multiple fibrotic changes in bilateral lung fields showing apicobasal gradient, with (D) honeycombing pattern and (B) patchy areas of consolidation and thickening of the septa in bilateral lung fields

